# Enhancing detection accuracy via controlled release of 3D-printed microlattice nasopharyngeal swabs

**DOI:** 10.1038/s44172-024-00185-5

**Published:** 2024-03-04

**Authors:** Ran Xiao, Jiaheng Li, Yue Fung Wong, Lok Ting Chu, Yingxin Zhu, Liqiang Wang, Minghui Wu, Dingkun Zhang, Meng Gong, Joseph Lai, Kannie W. Y. Chan, Rong Fan, Ting-Hsuan Chen, Yang Lu

**Affiliations:** 1 Department of Mechanical Engineering, City University of Hong Kong, Kowloon, Hong Kong SAR PR China; 2 Nano-manufacturing laboratory (NML), Shenzhen Research Institute of City University of Hong Kong, Shenzhen, 518057 PR China; 3 Department of Biomedical Engineering, City University of Hong Kong, Kowloon, Hong Kong SAR PR China; 4 Laboratory of Clinical Proteomics and Metabolomics, Institutes for Systems Genetics, Frontiers Science Center for Disease-related Molecular Network, National Clinical Research Center for Geriatrics, West China Hospital, Sichuan University, Chengdu, PR China; 5 Chengdu Research Institute of City University of Hong Kong, Chengdu, 610200 PR China; 6 Department of Mechanical Engineering, The University of Hong Kong, Pokfulam, Hong Kong SAR PR China

**Keywords:** Mechanical engineering, Biomedical engineering

## Abstract

Nasopharyngeal (NP) swab is one of the most effective sampling devices for clinical specimens. However, commercial NP swabs often release samples through diluents, lowering analyte concentration and causing inaccurate detections. Here, we developed 3D-printed open-cell microlattice NP swabs with user-friendly high-efficiency controlled sample release (CR) mode. Compared with traditional NP swabs, our microlattice NP swabs show higher (~7–11 times) flexibility, larger (~2.3 times) and customizable release volume, higher (dozens to thousands of times) release concentration, high recovery efficiency (~100%), and the ability to quantify analyte levels. Our microlattice NP swabs have been thus demonstrated to improve the sensitivity and accuracy of antibody detection experiments using rapid detection kits. This study offers a promising approach to enhance sensitivity and accuracy in clinical specimen detections, and is beneficial to inspire the design of a wider range of biomedical devices based on 3D-printed microlattice metamaterials.

## Abstract (web summary)

Ran Xiao, Jiaheng Li and colleagues report the fabrication of architected microlattice nasopharyngeal swabs, which can effectively release the collected load without the need for dilution. The method was demonstrated able to improve the accuracy of Covid19 rapid tests.

## Introduction

Nasopharyngeal (NP) swabs are one of the most effective sampling devices for clinical specimen detection. For example, during the COVID-19 pandemic, they are widely utilized in SARS-CoV-2 virus and antibody detection sampling, and play a vital role in preventing the spread of the epidemic. Collecting NP mucus using NP swabs and detecting it through medical laboratory testing or point-of-care testing (POCT)^1,2^ has become our very familiar daily routine. However, especially in POCTs, the commercial NP swabs often release samples through elution buffer (diluted release, DR), which greatly dilutes the analyte. Additionally, commercial NP swabs often show unsatisfactory recovery efficiency (sample release ratio from the swabs to the elution buffer^3^), which further reduces the analyte concentration. According to Bruijns et al., when collecting saliva samples through cotton, foam, nylon flocked, polyester, and rayon swabs for pure DNA isolation, more than 50% of the DNA remained in each type of swabs^3^. The existing POCTs such as rapid test kit detections often have limited sensitivity and unevenly varied quality. Low analyte concentrations greatly affects their detection accuracy^3–8^ leading to inaccurate detection results such as slow positives and false negatives^9^. Improving the sensitivity of the detection methods is undoubtedly the most direct way to improve detection accuracy. However, this idea often leads to heavy workload, large costs, long R&D cycles, and limited improvement. Beginning with the crucial sample release procedure, this challenge can be addressed more effectively if a new technique is created to break the concentration limits of sample release.

3D printing technologies provide a new method to solve the supply shortage of commercial NP swabs in the COVID-19 pandemic^10,11^. The original 3D-printed NP swabs designed with solid structures with micro convex arrays were verified to be statistically comparable and even replaceable to the commercial NP swabs in sampling effectiveness^10–24^. Compared with the commercial NP swabs of standardized specifications, they can be designed with individualized size and shape to better fit different individuals or various sampling sites^1,20,25^ (Supplementary Fig. S1). Researchers further introduced microlattice structures to optimize the sampling ability of the 3D-printed NP swabs, and demonstrated their multiple advantages^22–30^. For example, the large surface area and unit cell space of the microlattice structures can make the samples easier to be captured and retained. Their good flexibility contributes to better conform to the contours of the nasal cavity. Their capillary action help fasten sample collection. Their high energy absorption capacity can better resist the impact of the NP swab in the process of extending into the nasal cavity. Their mechanical performances and sampling capability can also be further customized through structural optimization. However, these advantages can hardly be regarded as irreplaceable advantages of 3D-printed NP swabs over traditional NP swabs. Especially when the shortage of the commercial NP swabs is gradually alleviated, and their widespread and effective service in practical sampling is resumed.

Here, via truss-based mechancial metamaterial concept, we propose 3D-printed microlattice NP swabs with optimized design and unignorable advantages over traditional NP swabs. Unlike current 3D-printed lattice NP swabs which are more like cylindrical structures with hollow surface, our microlattice NP swabs are fully filled with 3D open-cell microlattices. Compared with the commercial NP swabs, our microlattice NP swabs shows better flexibility (up to ~11 times), higher (~2.3 times) and customizable sample release volume, and are able to quantify the analyte level. More important, an easy and efficient controlled release (CR) method was also developed based on their geometry features. The CR method involves volume-controllably separating liquid from a microlattice NP swab using centrifugal force (manually or using a centrifuge) into the bottom of the swab container such as a centrifuge tube. It not only maintains the original concentration of the collected samples without relying on any complex equipment, but also realizes a high recovery efficiency (~100%), breaking through the concentration limitation of the released samples through traditional ways. Additionally, through an antibody detection experiment via rapid detection kit, our microlattice NP swab with undiluted CR ability was proved to show great potential to improve the detection sensitivity and accuracy of the detection system. These results suggest that our microlattice NP swabs are likely to be the secret weapon to widely improve the sensitivity and the accuracy of practical detections.

## Results and discussion

### Design and manufacturing

**Fig. 1:**
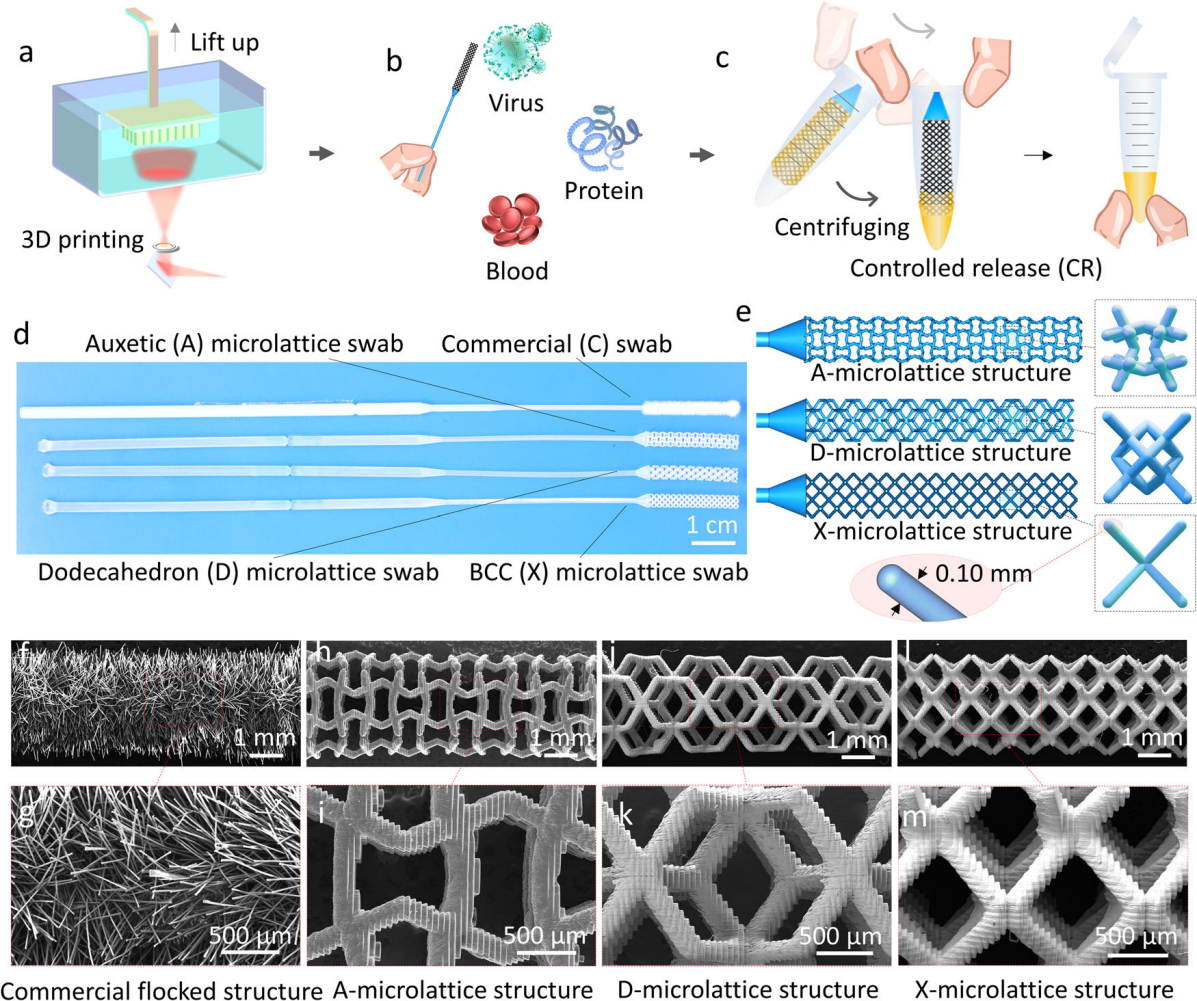
Design and manufacturing of the microlattice NP swabs. **a** Productive 3D printing of microlattice NP swabs; **b** Potential multi-substance sampling of the microlattice swabs; **c** Controlled release (CR) process of a microlattice swab through manual centrifuging; **d** 3D-printed open-cell Auxetic (A) microlattice NP swab, Dodecahedron (D) microlattice NP swab, and BCC (X) microlattice NP swab and the commercial flocked NP swab; **e** CAD models and geometry details of the 3D-printed A, D, X microlattice NP swabs; **f**–**m** and their SEM images.

The design scheme and manufacturing process of the 3D printed open-cell microlattice NP swabs are shown in Fig. 1. As shown in Fig. 1a, arranging and printing multiple swabs at once can reduce the ratio of the overall height to cross-sectional area of the printed model (as shown in Supplementary Fig. S1a). This can improve the printing success rate of slender structures like swabs. Clinical specimens are collected with the microlattice NP swabs (Fig. 1b), and then controllably released through centrifugal force (manually or using a centrifuge, Fig. 1c). Figure 1d shows three complex 3D open-cell microlattice NP swabs (the Auxetic (A) microlattice NP swab, Dodecahedron (D) microlattice NP swab, BCC (Body Center Cubic structure, abbreviated as X) microlattice NP swab) and the commercial flocked swabs, which are of similar overall shapes and dimensions. Figure 1e shows the design details of the 3D printed open-cell microlattice NP swabs. Their geometric details such as strut diameter, surface area, volume, specific surface area (the ratio of surface area to volume), porosity, and overall dimensions are listed in supplementary Table 1 and Supplementary Fig. 1d. Figure 1f–m shows the SEM images of the commercial flocked NP swab and the A, D, X microlattice NP swabs, suggesting good 3D printing qualities. Compared with the staggered fiber structures of the commercial NP swab, the large open-cell structures of our microlattice NP swabs show the potential of easier sample capture and target release, and less fluid retention inner the swab after sample release.

### Mechanical performance characterization

**Fig. 2:**
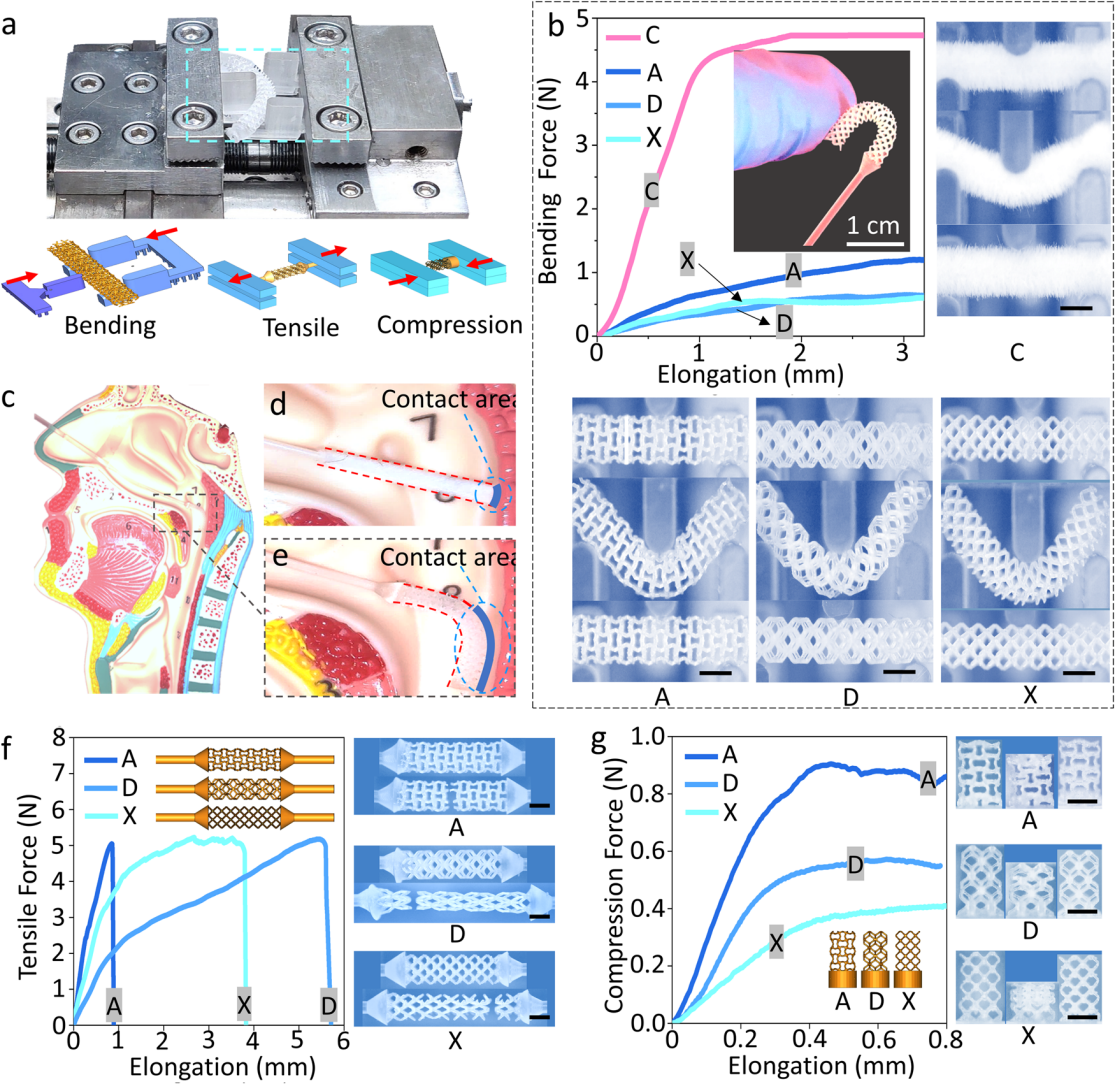
Mechanical characterizations of commercial flocked swab (C), Auxetic microlattice NP swab (A), Dodecahedron microlattice NP swab (D), and BCC microlattice NP swab (X). **a** Micro testing system and the schematics of the bending, tensile, and compression experiments; **b** Bending results and in-situ deformation processes of the commercial flocked NP swab and the 3D-printed A, D, X microlattice NP swabs; **c**–**e** Microlattice NP swabs are of better flexibility and bigger contact area with the NP cavity than the commercial ones; **f** Tensile results and in-situ deformation processes of the A, D, X microlattice NP swabs; **g** Compression results and in-situ deformation processes of the A, D, X microlattice NP swabs. (scale bars in **b**, **f**, and **g** are 2 mm).

To characterize the mechanical performances of the microlattice NP swabs, bending, tensile, and compression tests were performed with a micro testing system, as shown in Fig. 2a. Flexibility is essential for NP swabs as they must bend and penetrate far into the nasopharyngeal cavity to collect sufficient samples^22,26,31,32^. Figure 2b demonstrates that the reactive bending force (platform force) of the microlattice NP swab is up to $\sim \!$7 times less than that of the commercial NP swabs, and the flexibility (the reciprocal of the slope) of the microlattice NP swabs are up to ~11 times of that of the commercial NP swab. Figure 2b also shows the bending deformation process of the commercial NP swab and the A, D, X microlattice NP swabs from the pristine state, to maximum deflection state, and then to the unloaded state. Both the commercial NP swab and the microlattice NP swabs recovered their original shape after unloading, showing good resilience. The maximum deflection of the former was much smaller than that of the latter, also suggesting better flexibility of the latter. Owing to the good flexibility of the microlattice structure, our NP swab could have a better fit and a larger sampling area inside the cavity compared to the commercial swab, which is beneficial to increase the sampling volume (Fig. 2c–e). Additionally, better flexibility allows the microlattice NP swab head moving freely through the nasal cavity and exerting substantially less pressure on the surrounding tissue, showing the potential to reduce the discomfort and pain of patient.

During sampling, NP swabs also experience forces such as extrusion, friction, and collision with the NP cavity^15,32–34^. Here, tensile and compression tests were also performed. Figure 2f, g shows the force-elongation curves and the in-situ deformation processes of tensile tests and compression tests of the A, D, X microlattice structures, respectively. According to Park et al. who measured the applied force during the NP swab sampling procedure, the average maximum peak force on the z-axis is 0.596 N, on the x-axis is 0.217 N, and on the y-axis is 0.291 N^34^. According to Fig. 2f, during tensile process, the A, D and X structures exhibit similar tensile strength ($\sim \!$N), while their fracture strain decrease in the order of D, X, and A, indicating the decreasing trend of their structural toughness. Similarly, seen from Fig. 2g, the compressive strength decreased in the order of A ($\sim$0.9 N), D ($\sim$0.55 N), and X ($\sim$0.4 N). These results suggest that the designed microlattice structures are suitable swab tip structures which are strong enough to enter and exit the nasal cavity for sampling. Additionally, their mechanical performances can be easily adjusted and customized through changing the microlattice’s structural parameters such as the strut diameter, the cell size, and so on^35–38^. In future research, we can further design, optimize, and customize the microlattice structures of a swab based on more comprehensive mechanical data.

### Quantitative measurement of release concentration and release volume

**Fig. 3:**
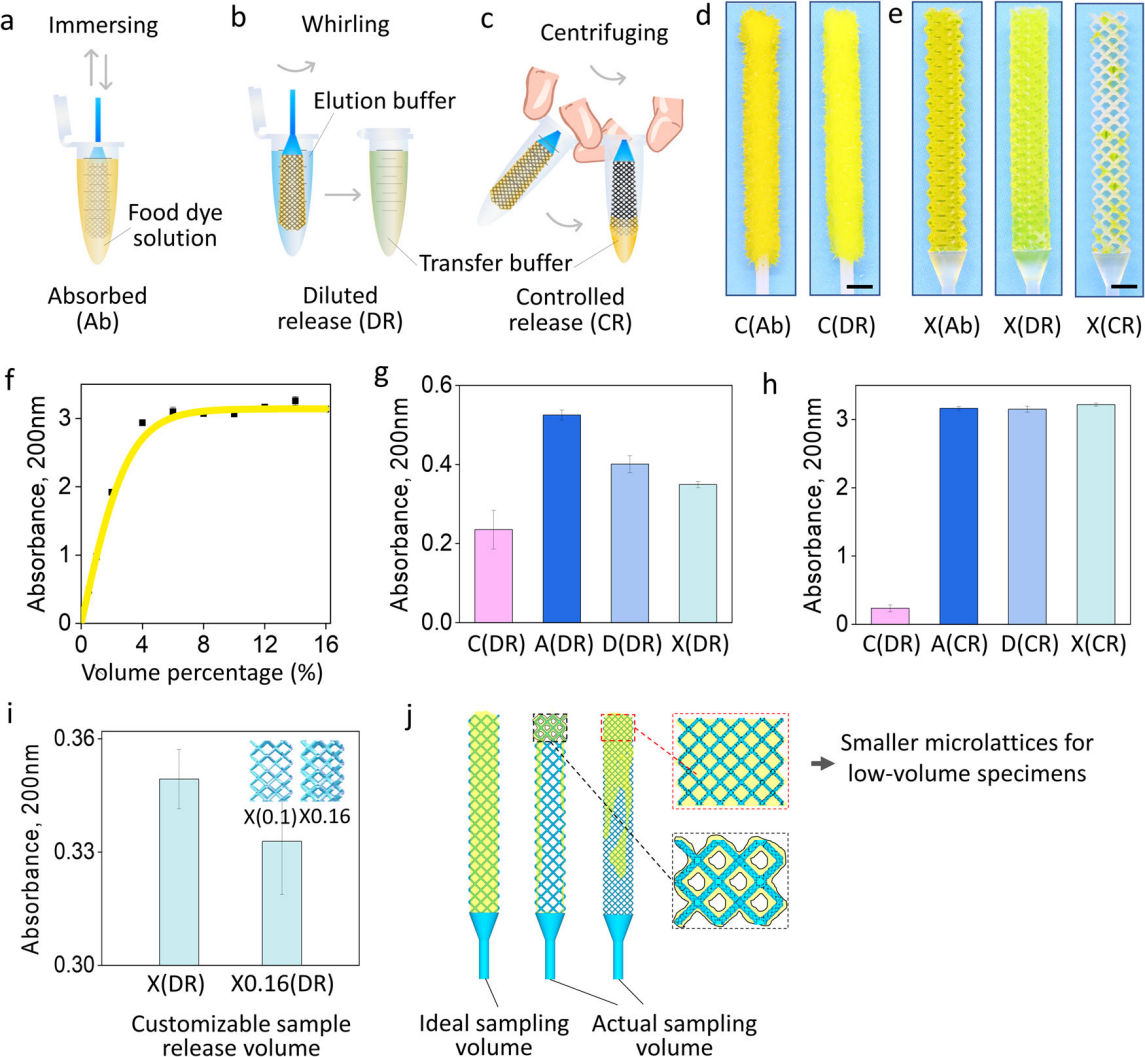
Releasing capability comparison of the 3D-printed Auxetic microlattice NP swab (A), Dodecahedron microlattice NP swab (D), and BCC microlattice NP swab (X) and the commercial flocked NP swab (C). **a** Schematic of sample (food dye solution) absorption of a NP swab; **b** Schematic of the diluted release (DR); and the **c** Controlled release (CR); **d** Commercial flocked NP swab absorbed with food dye solution and after DR; **e** 3D-printed X-microlattice NP swab loaded with food dye solution and after DR and CR, respectively; **f** Standard curve of food dye concentration (volume percentage) – food dye solution absorbance; **g** Food dye transfer buffer absorbances of the C(DR), A(DR), D(DR), and X(DR) (mean ± SD, *n* = 3); **h** Food dye transfer buffer absorbances of the C(DR), A(CR), D(CR), and X(CR) (mean ± SD, *n* = 3); **i** Food dye transfer buffer absorbances of X-microlattice NP swabs with strut diameters of 0.1 mm and 0.16 mm (abbreviated as X and X0.16, respectively), after DR (mean ± SD, *n* = 3); **j** Microlattice NP swabs with smaller microlattice cells are useful for sampling dilute or low-volume samples. (scale bars in **d** and **e** are 2 mm).

To compare the sample release ability of the microlattice NP swabs with commercial flocked NP swabs, two sample release ways are named. Initial food dye solution with a 1:9 volume ratio of yellow food dye to DI water were prepared as the specimens to be collected. The samples are first loaded into the NP swabs (Fig. 3a). Diluted release (DR) means transfer the samples from the swabs into the elution buffer (pure buffer before sample release without containing analyte) through manually agitating (Fig. 3b). The controlled release (CR) method involves volume-controllably separating liquid from a microlattice NP swab using centrifugal force (manually or using a centrifuge) into the bottom of the swab container such as a centrifuge tube under the action of centrifugal force (Fig. 3c)^39^. For a comparison of the two release methods, see Supplementary Movie 1. The CR mode can maintain the original concentration of the sample. Meanwhile, the sample release amount (volume) can be controlled by multiple ways, such as customizing the open space volume of the swab head and adjusting the centrifugal force. Figure 3d shows the commercial NP swab absorbed with initial food dye solution (C(Ab)) and then released through DR method (C(DR), in 3 mL DI water). Figure 3e shows the X- microlattice NP swab loaded with initial food dye solution (X(Ab)) and then release sample through DR method (X(DR), in 3 mL DI water) and CR method (X(CR)), respectively. According to Fig. 3d, e, swabs after DR exhibit much food dye retention, while the swabs after CR show minor food dye retention. The large opening space of the microlattice facilitates the entry and exit of samples, coupled with the CR under the action of centrifugal force, it can release the sample thoroughly, resulting in a small amount of sample residue, and achieving high recovery efficiency which is very competitive with traditional cotton swabs^3^.

Figure 3f gives the standard curve which reveals the correspondence between the food dye concentration (volume percentage) in a food dye solution and the absorbance of that food dye solution (see Supplementary Note 1 for the fitting formula). Figure 3g shows the absorbance measurement results of the food dye transfer buffers (as shown in Fig. 3b, c, for DR, transfer buffer is the elution buffer contains analyte after sample release. For CR, transfer buffer is the sample released by the swab) of both the commercial NP swab and the microlattice NP swabs after DR. Figure 3h exhibits the absorbance measurement results of the food dye transfer buffers of the commercial NP swabs after DR (in 3 mL DI water) and the microlattice NP swabs after CR. According to Fig. 3g and the standard curve in Fig. 3f, the food dye concentrations (volume percentage) of the commercial NP swab and the A, D, X-microlattice NP swabs after DR are 0.23%, 0.52%, 0.40%, 0.35%, respectively. The released food dye concentrations of the microlattice NP swabs are$\sim \!$1.5–2.5 times of that of the commercial NP swabs. Meanwhile, the the food dye concentrations (volume percentage) of the 3D printed microlattice NP swabs after CR are$\sim \!$10% (the food dye concentration of the initial food dye solution is 10%), showing$\sim \!$50 times of that of the commercial NP swabs after DR.

According to Fig. 3g, f, the volumes of food dye as well as the initial food dye solution released by the commercial NP swab and the microlattice NP swabs through DR (in 3 mL DI water) can also be quantitively calculated out (see Supplementary Note 1 for the calculation procedure)^40^. The volume of food dye released into the transfer buffer (${V}_{{fd}}$) by the NP swabs after DR are: ${V}_{{fd}-C({DR})}\approx 7.1\,{{{{{\rm{\mu L}}}}}}$, ${V}_{{fd}-A({DR})}\approx 16.5{{{{{\rm{\mu L}}}}}}$, ${V}_{{fd}-D({DR})}\approx 12.5{{{{{\rm{\mu L}}}}}}$, ${V}_{{fd}-X({DR})}\approx 10.9{{{{{\rm{\mu L}}}}}}$, which can quantify the analyte (food dye here) amount released. In practical detections, it is beneficial for better understanding the analyte level in human body^41^. The volume of initial food dye solution released into the transfer buffer (release volume, ${V}_{r}$) by the NP swabs after DR are: ${V}_{r-C({DR})}\approx 70.6{{{{{\rm{\mu L}}}}}}$, ${V}_{r-A({DR})}\approx 164.6{{{{{\rm{\mu L}}}}}}$, ${V}_{r-D({DR})}\approx 125{{{{{\rm{\mu L}}}}}}$, ${V}_{r-X({DR})}\approx 108.8{{{{{\rm{\mu L}}}}}}$, which can reflect the sample storage/release capacities of the NP swabs. It can be seen that the release volume of the microlattice NP swabs is up to $\sim \!$2.3 times of that of the commercial one, reflecting superior sample storage/release capacities of our 3D printed microlattice NP swabs. Additionally, by designing the microlattice structure, we can quantitatively customize the sampling amount of the swab. For example, Fig. 3i shows the absorbances of the food dye transfer buffers of X-microlattice NP swabs with different truss diameters after CR. The different truss diameters of the X microlattice structures lead to the difference in the open-cell sample storage volume of the swab tips, which ultimately leads to different sample release volumes. In addition, if the samples to be collected is diluted or low-volumed, it is recommended to design the microlattice swabs with smaller cell sizes and strut diameters such as micro or nano microlattice, which can provide better sample storage (Fig. 3j). It is worth noting that, as shown in Fig. 3j, due to insufficient samples in practice which often cannot fully fill the entire swab tip, the released sample volumes will be less than the experimental results. This would result in greater sample release concentration differences.

We can see that whether through DR method or undiluted CR method, the microlattice NP swabs show much better sample release performances than the commercial flocked one. On the one hand, through CR, retaining the original concentration of the collected samples makes 3D-printed microlattice swabs have unique advantages over traditional swabs in certain scenarios. For example, the detection site/individual with low analyte level^6,42^. It would assist in broadly improving the sensitivity, accuracy and tolerance of the detection methods. On the other hand, according to Jun et al., the open-fiber structure of nylon flocked swabs leading to better sample release^4,43^. The same causality applies to microlattice swab’s larger open cell structure and its consequent higher recovery efficiency. According to Fig. 1f–m, compared with the crossed fiber structures of the flocked NP swabs with entangled microfibers^1,44^, 3D-printed NP swabs with three-dimensional open cell microlattice structures are of larger unit cell and more open space. During DR, as the microlattice NP swabs retain and contain samples through the space of open-cell structures, the samples are easier to be captured and released, and less sample will be retained after release. When further centrifuging out the retained liquid from the microlattice, an excellent sample recovery efficiency can be achieved, which is competitive with both traditional fiber structured swabs and other types of 3D-printed swabs^1,4,5,7,8^.

### Viscous liquid sampling and quantitative release

**Fig. 4:**
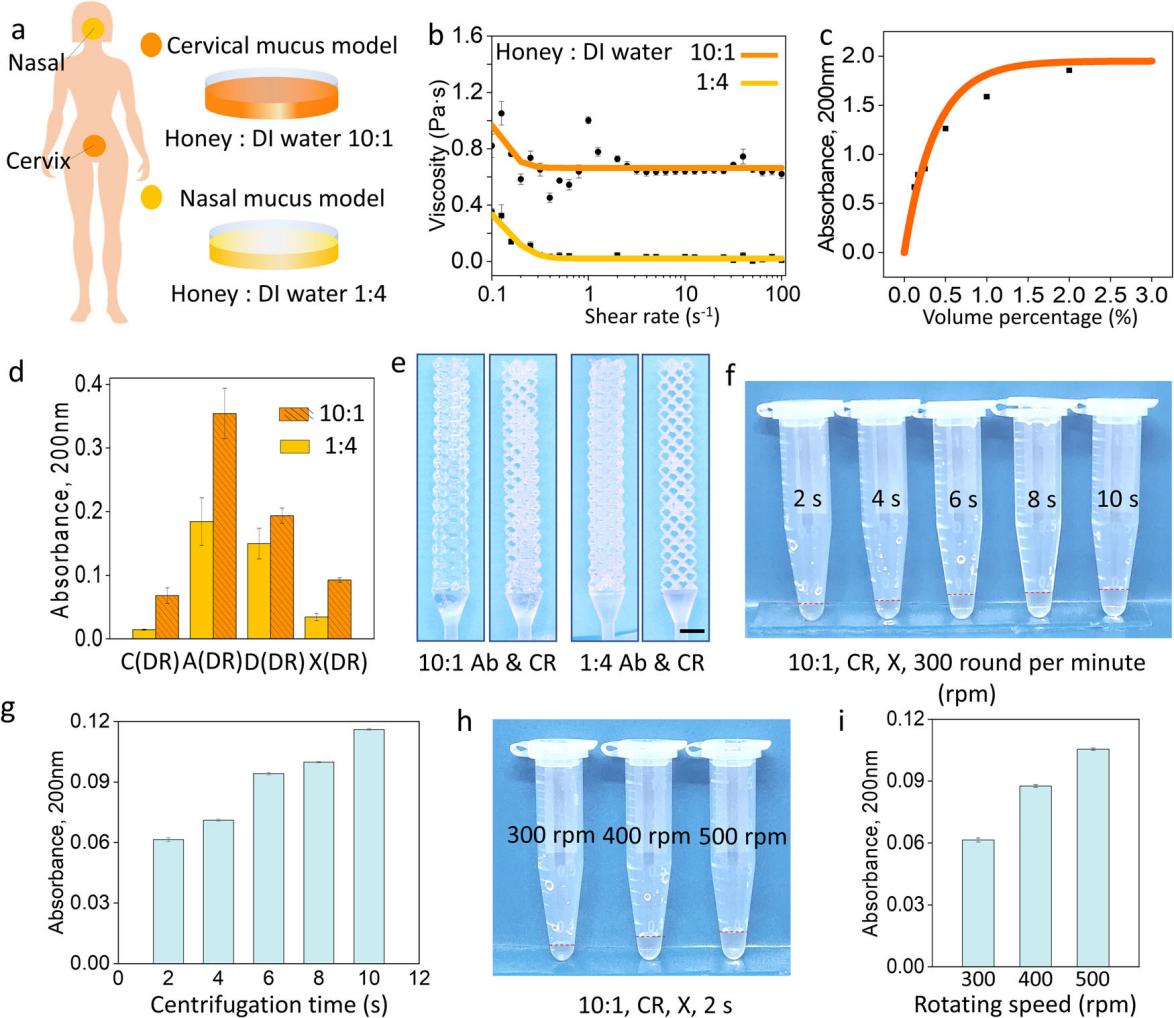
Viscous liquid sampling and quantitative release demonstration. **a** Human cervical mucus model and nasal mucus model made of honey solutions with honey to water ratio of 10 to 1 and 1 to 4, respectively; **b** Viscosity-shear rate curves of the cervical mucus model and nasal mucus model (dark orange line with round symbol for 10:1 honey water, and light orange line with square symbol for 1:4 honey water); **c** Standard curve of honey concentration (volume percentage) – honey solution absorbance; **d** Honey transfer buffer absorbances of the C(DR), A(DR), D(DR), and X(DR) (mean ± SD, *n* = 3); **e** Qualitative demonstration of X-microlattice NP swabs release cervical mucus model and nasal mucus model through CR (scale bar: 2 mm); X-microlattice NP swab’s quantitative sample release ability realized by a centrifuge through adjusting the rotation speed (**f** and **g**) and the duration (**h** and **i**) of the centrifuge (in **g** and **i**, mean ± SD, *n* = 3; A 3D-printed Auxetic microlattice NP swab, D Dodecahedron microlattice NP swab, X BCC microlattice NP swab, and C the commercial flocked NP swab, Ab absorbed, DR diluted release, CR controlled release).

To better simulate the sampling situation in reality, we use honey solutions as mucus models to further know the sample capture and target release ability of our microlattice NP swabs for human mucus^26^. Initial honey solutions with honey to water ratio of 10 to 1 and 1 to 4 were made to mimic human cervical mucus and nasal mucus, respectively (Fig. 4a). Figure 4b shows their shear rate-viscosity curves which are comparable to human cervical mucus and nasal mucus^5,45^. Figure 4c gives the standard curve which reveals the correspondence between the honey concentration (volume percentage) in a honey solution and the absorbance of that honey solution (see Supplementary Note 2 for the fitting fomula). We investigated the sample release performance of microlattice NP swabs and commercial NP swabs on these mucus models with different viscosities via DR (Fig. 4d, quantitatively) and via CR (Fig. 4e, qualitatively). Figure 4d shows the absorbance measurement results of the honey transfer buffers (in 3 mL DI water) of both the commercial NP swab and the microlattice NP swabs after DR. According to Fig. 4d, the DR ability of the A-microlattice NP swab is better than that of the D-microlattice NP swab and the X-microlattice NP swab.

According to Fig. 4d and the standard curve in Fig. 4c, for the sampling of cervical mucus models, the honey concentrations (volume percentage) of the commercial NP swab and the A, D,-microlattice NP swabs after DR are 0.06%, 0.33%, 0.17%, and 0.08%, respectively. The released honey concentrations of the microlattice NP swabs are up to $\sim$6 times of that of the commercial NP swabs. Meanwhile, the honey concentrations (volume percentage) of the 3D printed microlattice NP swabs after CR are $\sim$90% (the honey concentration of the initial honey solution is $\sim \!$90%), showing $\sim \!1$500 times of that of the commercial NP swabs after DR. For the sampling of nasal mucus models, the honey concentrations (volume percentage) of the commercial NP swab and the A, D, X -microlattice NP swabs after DR are 0.013%, 0.17%, 0.14%, and 0.03%, respectively. The released honey concentrations of the microlattice NP swabs are up to $\sim \!$13 times of that of the commercial NP swabs. Meanwhile, the honey concentrations (volume percentage) of the 3D printed microlattice NP swabs after CR are $\sim$20% (the honey concentration of the initial honey solution is 20%), showing $\sim 1$500 times of that of the commercial NP swabs after DR. Likewise, due to insufficient samples in practice, less released samples lead to greater sample release concentration differences. For the sampling of viscous liquid samples, using microlattice NP swabs is considered superior to commercial NP swabs. The higher the viscosity of the sample, the more pronounced this advantage becomes.

Figure 4e shows the CR results of the microlattice NP swab for two mucus models under the same centrifuging force. Figure 4f–i shows the X-microlattice NP swab’s quantitative sample release ability realized by a centrifuge through adjusting the rotation speed (Fig. 4f, g) and the duration (Fig. 4h, i) of the centrifuge. Apparently, the X-microlattice NP swab absorbed the higher viscosity cervical mucus model centrifuged out less samples and remained more samples on the swab. In addition, the microlattice NP swabs were demonstrated to show quantitative sample release abilities which can be realized through multiple ways. On the one hand, the open-cell 3D spaces of the microlattice structures for sample accommodating can be customized by changing their geometric features such as shapes (Figs. 3g and 4d) and sizes (Fig. 3i, j). On the other hand, adjusting the time and rotating speed of the centrifuge can also control the sample volumes undilutedly released by the microlattice NP swabs. Figure 4f, h shows the obtained quantitative honey transfer buffers of the X-microlattice NP swabs after CR. The quantitative CRs were realized by adjusting the duration and the rotating speed of a centrifuge, respectively. Figure 4g, i gives the absorbances of the honey transfer buffers diluted with 3 mL of DI water in Fig. 4f, h, respectively. The centrifugal force formula is as follows:

5 $${{{{{\rm{F}}}}}}={{{{{\rm{m}}}}}}{{{{{{\rm{\omega }}}}}}}^{2}{{{{{\rm{r}}}}}}={{{{{\rm{m}}}}}}{(2{{{{{\rm{\pi }}}}}}{{{{{\rm{n}}}}}})}^{2}{{{{{\rm{r}}}}}}$$

Where F is the centrifugal force, ω (rad/s, ω = rpm*$2{{{{{\rm{\pi }}}}}}$/60) is the angular velocity (With the help of the angular velocity sensor measurement, we obtained that the ω of a manual centrifuging is ~35 rad/s, while for the situations in Fig. 4, the corresponding ω of 300 RPM, 400 RPM, and 500 RPM are 31.4, 41.9, and 52.4 rad/s, respectively), and n (${s}^{-1}$) is the rotating speed. Whether CR of microlattice NP swabs is performed manually or through a centrifuge depends on the sample volume collected and whether quantitative release is required. If low-volume sample is collected (a bigger force is needed to swung it out), or an accurate release amount is required, a centrifuge should be used for CR. Otherwise, CR via manual centrifuging is enough.

### Demonstration of rapid test kit antibody detection

**Fig. 5:**
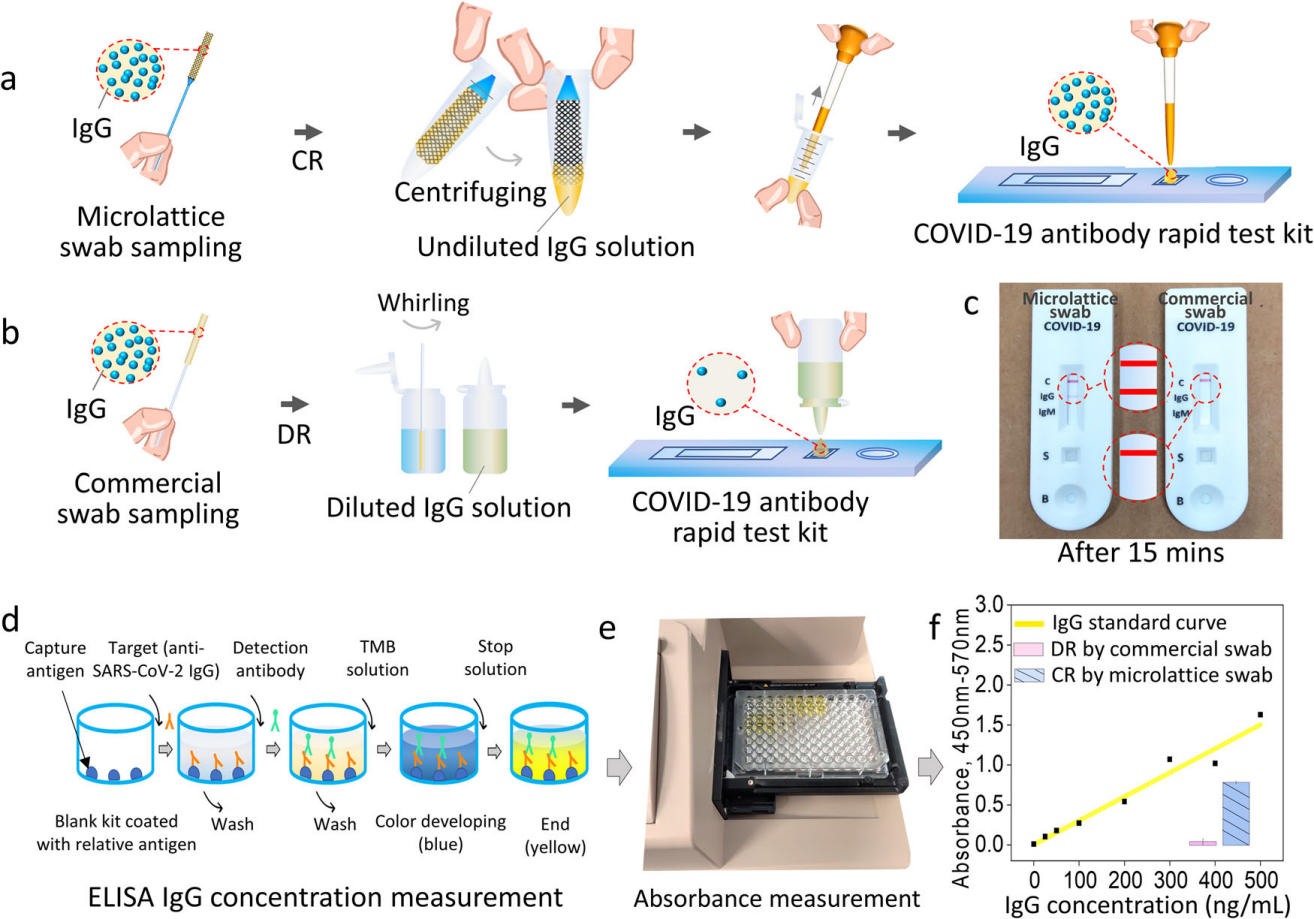
Demonstration of rapid test kit antibody detection. **a** Undiluted CR of anti-SARS-CoV-2 IgG specimen of 3D-printed microlattice NP swabs; **b** Diluted anti-SARS-CoV-2 IgG sample release with commercial flocked NP swabs; **c** Rapid test kit anti-SARS-CoV-2 IgG detection results of **a** and **b**; **d** Mechanism of ELISA; **e** Absorbance measurement of anti-SARS-CoV-2 IgG solutions in the ELISA kit; **f** Standard curve of anti-SARS-CoV-2 IgG concentration (ng/mL) – anti-SARS-CoV-2 IgG solution absorbance, and the absorbances of the anti-SARS-CoV-2 IgG transfer buffers of the two kinds of NP swabs in **a** and **b**. DR diluted release, CR controlled release.

As a demonstration, we applied the anti-SARS-CoV-2 antibody (IgG/IgM) rapid test kits and SARS-CoV-2 IgG Enzyme linked immunosorbent assay (ELISA) kit to detect the anti-SARS-CoV-2 IgG^46^ released by microlattice NP swabs via CR and by commercial NP swabs via DR. A-microlattice NP swab was chosen as a representative of microlattice NP swabs for this demonstration due to its best sampling performance, according to Figs. 3g and 4d. Initial anti-SARS-CoV-2 IgG solution with anti-SARS-CoV-2 IgG concentration of 300 ${ng}/{mL}$ was prepared and loaded into both the microlattice NP swabs and the commercial flocked NP swabs. The anti-SARS-CoV-2 IgG transfer buffer of the microlattice NP swabs after CR is collected as shown in Fig. 5a. The anti-SARS-CoV-2 IgG transfer buffer of the commercial NP swabs after DR (in 3 mL elution buffer (phosphate-buffered saline, 1% Tween 20®, PBST)) is collected as shown in Fig. 5b. Then, the anti-SARS-CoV-2 IgG transfer buffers from both the microlattice NP swabs and the commercial NP swabs were dropped onto the anti-SARS-CoV-2 antibody (IgG/IgM) rapid test kits. Figure 5c shows the detection results: the rapid test kits loaded with anti-SARS-CoV-2 IgG transfer buffers obtained by the microlattice NP swabs (after CR) showed positive results, verifying the presence of IgG (See Supplementary Movie 2). However, the rapid test kits loaded with anti-SARS-CoV-2 IgG transfer buffers obtained by the commercial NP swabs (after DR) got negative results, due to low anti-SARS-CoV-2 IgG concentration.

Through an IgG color reaction generated by an ELISA (details please find from the method section, and the reagents and equipment used in ELISA IgG concentration measurement please find from the Supplementary Fig. 2), we further quantified the concentration difference between the anti-SARS-CoV-2 IgG transfer buffers from both NP swabs. Figure 5d exhibits the mechanism of the ELISA, and Fig. 5e shows the absorbance measurement of anti-SARS-CoV-2 IgG solutions in the ELISA kit. Figure 5f gives the standard curve of anti-SARS-CoV-2 IgG concentration – anti-SARS-CoV-2 IgG solution absorbance (see Supplementary Note 3 for the fitting formula), as well as the absorbances of the anti-SARS-CoV-2 IgG transfer buffers of the NP swabs in Fig. 5a, b. According to Fig. 5f, the anti-SARS-CoV-2 IgG concentration of the anti-SARS-CoV-2 IgG transfer buffer of the microlattice NP swab is $\sim \!300{ng}/{mL}$, while that of the commercial flocked NP swab is $\sim 5{ng}/{mL}$, showing a $\sim$60 times difference. In practice, low-volume samples would result in even larger concentration differences. Our microlattice NP swab with undiluted CR ability shows the potential to greatly improve the detection sensitivity and accuracy of the detection system. Meanwhile, through quantitative calculation procedure as shown in the food dye solution specimen section, we can also know the analyte level in patient’s body quantitatively. For example, testing of antibody level can help people better understand their immune status^42^.

## Conclusions

With the COVID-19 pandemic, sampling swabs have entered the field of vision of the general public. In fact, besides being used in coronavirus detection, swabs have a very wide range of uses, such as collect various human secretions and applied in disease screening, pathological examination and medicolegal expertise^1,25^. Here, based on the high-precision 3D printed microlattice structure^47,48^, we propose 3D printed microlattice NP swab with unique controlled release (CR) method. Their flexibilities are up to ~11 times of that of the commercial NP swab, resulting better fit and a larger sampling area inside the cavity, which is beneficial to increase the sampling volume and reduce the discomfort and pain of patient. They are of superior sample storage and release capacities and an ~2.3 times greater release volume than that of the commercial counterpart. Their open-cell structures exhibit a highly competitive efficiency in sample recovery. Their sample release amount (volume) can be controlled by multiple ways (such as customizing the open space volume of the swab head and adjusting the time and rotating speed of the centrifuge). Their sample concentration obtained through CR is at least dozens to thousands of times of that obtained by traditional swabs via DR (diluted release, DR), breaking through the concentration limitation of the released samples through traditional ways, which can effectively make up for the extensive detection misjudgment caused by the insufficient analyte titer caused by the traditional DR method. Additionally, the analyte amount released can be quantified through quantitative calculation. They are also demonstrated the potential to effectively improve the sensitivity and accuracy of detection systems in antibody detection experiments with rapid test kits. In future research, their mechanical performances and sample storage capacity can be easily customized through changing the microlattice structural parameters such as truss diameters.

As this article only takes food dye, honey, and IgG as samples for release experiments, the real clinical samples are more diverse and complex, which will lead to the limitations of this article. However, we believe that the data obtained so far are sufficient to demonstrate the unignorable advantages of microlattice NP swabs over traditional swabs. This paper fully based on the structural properties of microlattice metamaterials, to propose potential solutions to the hot issues in the field of biomedicine^49^. We believe this work will bring innovation and reference to the design of various biomedical devices based on 3D-printed microlattice metamaterials including various swabs.

## Methods

### Design and manufacturing

Here, the Auxetic (A), Dodecahedron (D), and BCC (X) microlattice swabs were modeled though Rhinoceros software. Supplementary Table 1 gives the detailed geometrical parameters of these structures. The 3D printing was performed by a high-resolution liquid-crystal display (LCD) 3D printer Whale 2 (Shenzhen Nova Intelligent Technology Co., Ltd.) using a commercial transparent tough ABS-like photosensitive resin (G217, RESIONE). For printing, the exposure time was set as 75 s for the first 8 layers and 6 s for the rest of layers, and the layer thickness was set as 50 μm. After printing, to remove residual resin, the samples were washed with ethanol and distilled water, and dried in a vacuum drying box with silica gel desiccant.

### Mechanical characterization

The bending, tensile, and compression tests on the microlattice swab tips or structures were conducted by a micro testing system (Gatan™ Microtest, shown in Fig. 2c). Tensile and compression tests were conducted directly on the micro testing system, while an individual bending device was specially designed and 3D-printed to attach onto the micro testing system for 3-point bending tests, as shown in Fig. 3b. Bending samples are the microlattice swab tips taken from the complete microlattice NP swabs. Considered the travel distance of the two ends of the micro testing system, the length of the tensile samples and compression samples were designed as $\sim$10 mm and $\sim \!$5 mm, respectively. To ensure the reproducibility of each experimental result, each design type of NP swab was tested more than three times in each mechanical test.

### Sample release characterization

Commercial yellow food dye and DI water were prepared in a volume ratio of 1–9 to make the initial food dye solution. Centrifuge tubes were prepared, and 1 ml of the food dye model solution was put into each centrifuge tube for sample absorption. 3D-printed microlattice NP swabs and commercial flocked NP swabs (SZ010, JLX Industrial Co., Ltd, Shenzhen) were prepared and immersed into each centrifuge tube for 10 s to fully load the model solution. The commercial flocked NP swab was placed into 3 mL DI water and stirred 20 times for fully DR. Part of the 3D-printed microlattice NP swabs were placed into 3 mL DI water and stirred 20 times for fully DR. Another part of the 3D-printed microlattice NP swabs was placed into the empty centrifuge tube and CR through manually or centrifuge shaking. The processes of DR of both flocked swab and microlattice swab and CR of the microlattice swab were shown in Supplementary Movie 1. An UV-Vis spectrometer (BioDrop μLITE, UK) was used to perform absorbance tests of the transfer buffers obtained after DR and CR. The absorbance result directly reflects the concentration (volume percentage) of food dye in the transfer buffer.

To simulate the sampling and release of viscous liquids, nasal and cervical mucus model solutions made of honey and DI water at volume ratios of honey to water as 1 to 4 and 10 to 1, respectively, were prepared. A rheometer (KINEXUS Pro+ (Malvern, UK) were applied to confirm the viscosities of these model solutions were within the nasal and cervical mucus viscosity ranges. A centrifuge (SORVALL LEGEND MICRO17, Thermo Scientific) was applied to exhibit the release volume customization ability of the 3D-printed microlattice NP swabs.

To ensure the reliability and robustness of the statistical analysis, for the sample release experiments, each experiment was repeated three times, while for each group of repeated experiments, three absorbance tests were performed and the average value was taken.

### Rapid test kit antibody detection demonstration

Anti-SARS-CoV-2 Spike RBD neutralizing antibody (anti-SARS-CoV-2 IgG, SAD-S35, Acro biosystems, abbreviated as IgG) and the anti-SARS-CoV-2 antibody (IgG/IgM) rapid test kits (COVID-19 IgM/IgG Antibody rapid test kit, BIOSYNEX, France) were used in the experiment of rapid test kit antibody detection demonstration. The IgG was reconstituted with sterile deionized water to a stock solution of 2000 µg/mL and gently mixed for 30–60 min at room temperature to solubilize. The solution was sub packed and stored at −80 °C for long term storage. The reconstituted stock solution was taken out and thawed before use. Stock solution was added in elution buffer (1 × Phosphate-Buffered Saline, 1% Tween 20®, PBST) to produce 10,000 ng/mL solution. 300 ng/mL IgG1 solution was made by diluting 10,000 ng/mL IgG solution in elution buffer. Two printed swabs and two commercial swabs were loaded with the solution in each tube and then released in another four tubes: two empty tubes were for the CR of the 3D-printed microlattice NP swabs, and the other two tubes were filled with 3 mL elution buffer for the DR of the commercial flocked NP swabs.

### ELISA IgG concentration measurement

ELISA aims at measuring the concentration difference of the IgG transfer buffers of the microlattice swab after CR and the flocked swab after DR in the rapid test kit antibody detection demonstration. The SARS-CoV-2 IgG ELISA kits (catalog no. ab275300, Abcam) were used in this section. The ELISA kit is pre-coated with antigens against anti-SARS-CoV-2 IgG. The targets (IgGs) were captured and remained by the antigens, and the extra substances were washed out. Then the detection antibodies were attached to the targets, and the extra substances were washed out. After adding TMB substrate, there happened a blue reaction. After adding stop solution, the color changed to yellow. The shades of the color were directly related to the titer of IgG, which would be quantified by subsequent absorbance testing. The transfer buffers of the 3D printed microlattice swabs (after CR) and the commercial swabs (after DR) were diluted 10 times and added into the appropriate wells before covering the wells with a sealing tape and incubating for 30 min at room temperature. The wells were washed 5 times with 250 µL of TNT wash buffer followed by adding 100 µL of detection antibody to each well. The plate was gently tapped for several times for mixing and incubated for another 30 min at room temperature. Then, the wells were washed 5 times with TNT wash buffer. After that, 100 µL of TMB substrate solution was added into each well and incubated in dark for 15 min at room temperature, the solution turned to blue. After 100 µL of stop solution was added into each well, the solution immediately turned to yellow. The OD was measured on a microplate reader set at 450 nm and 570 nm quickly after the reaction stopped. To make the detection more accurate, each experiment was performed in duplicate.

### Reporting summary

Further information on research design is available in the Nature Portfolio Reporting Summary linked to this article.

### Supplementary information

Supplementary Information

Description of Additional Supplementary Files

Supplementary Movie 1

Supplementary Movie 2

Reporting Summary

## Data Availability

Data supporting the conclusions of this work are available from the corresponding author upon reasonable request.
