# Supplementary material for: Enhancing detection accuracy via controlled release of 3D-printed microlattice nasopharyngeal swabs
Source: Commun Eng. 2024 Mar 4;3:40. doi: 10.1038/s44172-024-00185-5 (PMC10956077; doi:10.1038/s44172-024-00185-5)
Supplement: Supplementary file 1 — Supplementary Information [file 44172_2024_185_MOESM1_ESM.pdf]

Supplementary Information for

Enhancing detection accuracy via controlled release of 3D-printed microlattice nasopharyngeal swabs

The PDF file includes:

Supplementary Figure 1 and 2

Supplementary Table 1

Supplementary Data processing and Analysis

Legends for supplementary Movie 1 and 2

Other Supplementary Material for this manuscript includes the following:

Supplementary Movie 1 and 2

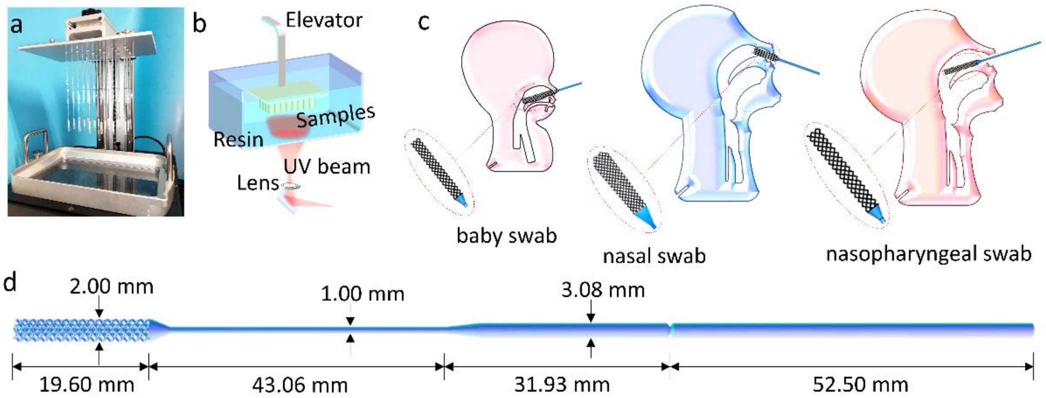

Supplementary Figure 1. a) Multiple swabs are printed simultaneously; b) Work principle of the LCD 3D printer; c) Customizable design of the microlattice NP swabs with different sizes, shapes, and types; d) The overall size details of a microlattice NP swab.

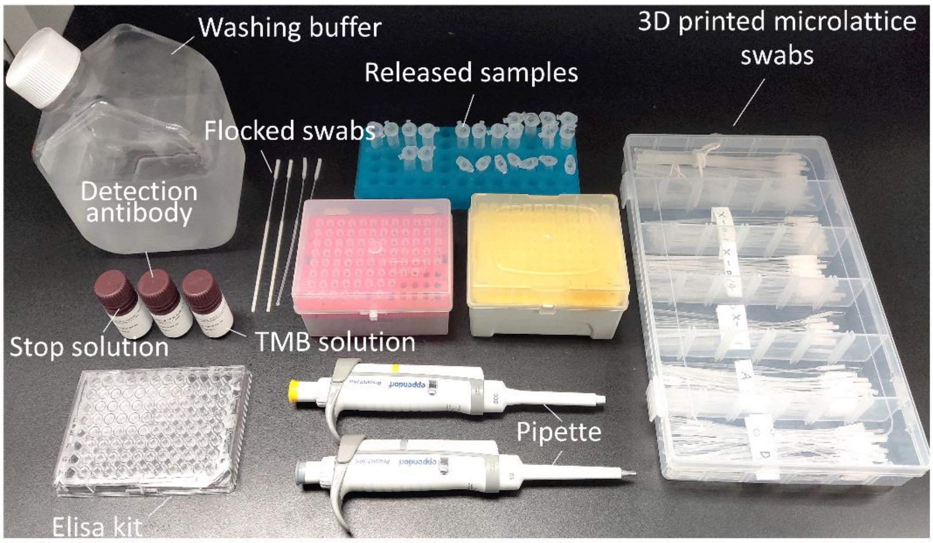

Supplementary Figure 2. Reagents and equipment used in ELISA IgG concentration measurement.

Supplementary Table 1. Detailed geometric features of the microlattice NP swab tips

Microlattice	Strut diameter (mm)	Cell size (mm)	Surface area (mm^2)	Volume (mm^3)	specific surface area (mm^{-1})	Porosity (%)
Auxetic	0.10	3.2	380.96	19.69	19.35	90.4
Dodecahedron	0.10	3.2	268.18	13.55	19.79	91.5
BCC(X)	0.10	3.2	350.62	17.01	20.61	88.8
BCC0.16(X0.16)	0.16	3.2	500.92	40.95	12.23	75.0

Supplementary Notes

- ### Supplementary Note 1

The standard curve formula in Figure 3f:

$$y = P_m \cdot \tanh\left(\frac{\alpha \cdot x}{P_m}\right) \quad (R^2 = 0.99848) \; (1)$$

where $x\%$ is the food dye concentration (volume percentage) in a food dye solution (for example, the transfer buffers of the NP swabs), and y is the relative absorbance of the food dye solution [1][2].

The food dye volume percentage in a transfer buffers after sample release can also be expressed as follows:

$$x\% = \frac{V_{fd}}{V_{tb}} \qquad (2)$$

where V_{fd} is the volume of food dye in the transfer buffer, and V_{tb} is the volume of the transfer buffer.

As

$$V_{fd} = a * V_r \qquad (3)$$

where a is the volume percentage of the food dye in the initial food dye solution (10% here). V_r is the release volume of the swab, which is the volume of the initial food dye solution released into the elution buffer from the swab, and

$$V_{tb} = V_r + V_{eb} \qquad (4)$$

where V_{eb} is the volume of the elution buffer (3mL here).

Thus, equation (2) is equivalent to:

$$x\% = \frac{V_{fd}}{\frac{V_{fd}}{a} + V_{eb}} \qquad (5)$$

which is equivalent to:

$$V_{fd} = \frac{a \times V_{eb} \times x\%}{\mathrm{a} - x\%} \qquad (6)$$

As a is 10% and V_{eb} is 3 mL (3000 µL), V_{fd} can be expressed as follows:

$$V_{fd} = \frac{300 \times x}{10 - x} \qquad (7)$$

We can thus get the the volume of food dye released into the transfer buffer by both the

commercial NP swab and the microlattice NP swabs after DR: $V_{fd-C(DR)} \approx 7.1$ μL, $V_{fd-A(DR)} \approx 16.5$ μL, $V_{fd-D(DR)} \approx 12.5$ μL, $V_{fd-X(DR)} \approx 10.9$ μL. And the release volume (V_r) of the NP swabs: $V_{r-C(DR)} \approx 70.6$ μL, $V_{r-A(DR)} \approx 164.6$ μL, $V_{r-D(DR)} \approx 125$ μL, $V_{r-X(DR)} \approx 108.8$ μL.

- **Supplementary Note 2**

The standard curve formula in Figure 4f:

$$y = -3.15 \times e^{-\frac{x\%}{2.77}} + 3.15 \quad (8)$$

where $x\%$ is the honey concentration (volume percentage) in a honey solution (for example, the transfer buffers of the NP swabs), and y is the relative absorbance of the honey solution.

- **Supplementary Note 3**

The standard curve formula in Figure 5f:

$$y = 0.003x + 0.0024 \quad (R^2 = 0.9645) \ (9)$$

where x is the anti-SARS-CoV-2 IgG concentration (ng/mL) in a anti-SARS-CoV-2 IgG solution (for example, the transfer buffers of the NP swabs), and y is the relative absorbance of the anti-SARS-CoV-2 IgG solution.

Supplementary References

[1] Chowdhury T, Benchimol S. Variability and inheritance of proteins in Apis mellifera eggs[J]. 2012.

[2] Zhang J X J, Hoshino K. Optical transducers: Optical molecular sensing and spectroscopy[J]. Mol. Sensors Nanodevices, 2019: 231-309.

Supplementary Movie 1. Process demo of release methods. The DR process of the commercial flocked NP swab, the DR process of the 3D printed microlattice NP swab, and the CR process of the 3D printed microlattice NP swab.

Supplementary Movie 2. Rapid test kit detection of anti-SARS-CoV-2 IgG transfer buffer released by the microlattice NP swab via CR and the commercial NP swab via DR. Only the transfer buffers of the 3D printed microlattice NP swabs after CR realize positive results, verifying the presence of anti-SARS-CoV-2 IgG. However, the transfer buffers of the commercial NP swabs after DR only got a negative result due to the low anti-SARS-CoV-2 IgG concentration. The undiluted CR of the 3D printed microlattice NP swabs shows the potential to greatly improve the sensitivity and accuracy of clinical specimen detections.
