# Supplementary material for: Enhancing detection accuracy via controlled release of 3D-printed microlattice nasopharyngeal swabs
Source: Commun Eng. 2024 Mar 4;3:40. doi: 10.1038/s44172-024-00185-5 (PMC10956077; doi:10.1038/s44172-024-00185-5)
Supplement: Supplementary file 2 — Description of Additional Supplementary Files [file 44172_2024_185_MOESM2_ESM.pdf]

Description of Additional Supplementary Files

File name: Supplementary Movie 1

Description: Process demo of release methods. The DR process of the commercial flocked NP swab, the DR process of the 3D printed microlattice NP swab, and the CR process of the 3D printed microlattice NP swab.

File name: Supplementary Movie 2

Description: Rapid test kit detection of anti-SARS-CoV-2 IgG transfer buffer released by the microlattice NP swab via CR and the commercial NP swab via DR. Only the transfer buffers of the 3D printed microlattice NP swabs after CR realize positive results, verifying the presence of anti-SARS-CoV-2 IgG. However, the transfer buffers of the commercial NP swabs after DR only got a negative result due to the low anti-SARS-CoV-2 IgG concentration. The undiluted CR of the 3D printed microlattice NP swabs shows the potential to greatly improve the sensitivity and accuracy of clinical specimen detections.
